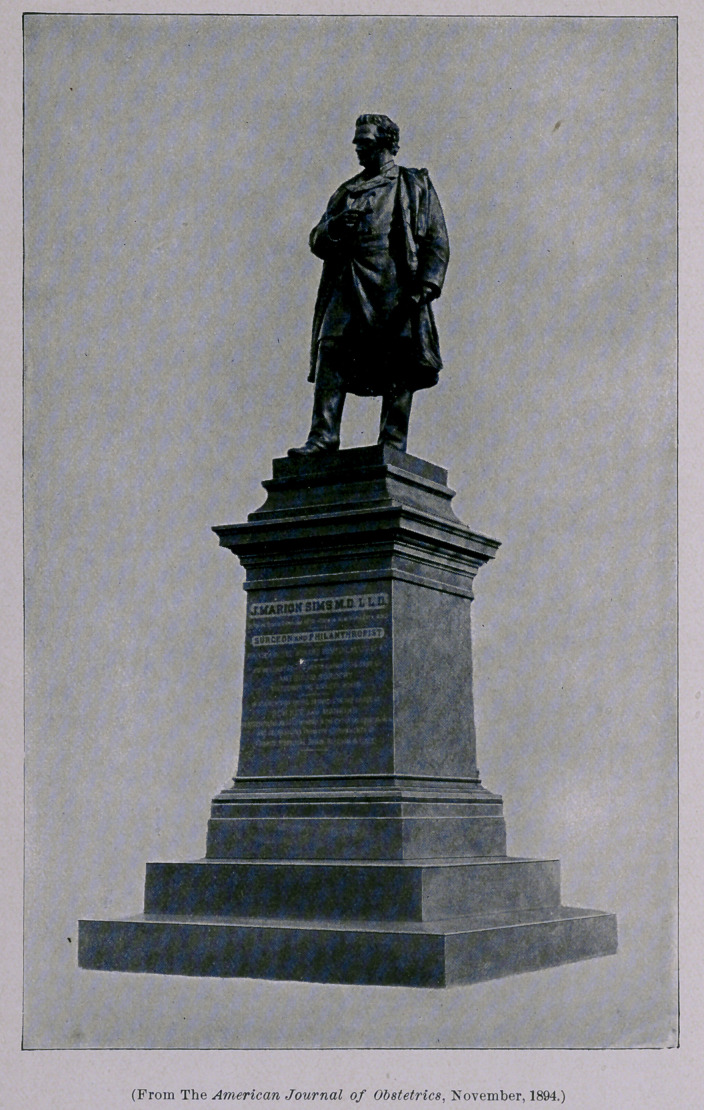# The Mississippi Valley Medical Association

**Published:** 1894-12

**Authors:** 


BUFFALO MEDICAL AND SURGICAL JOURNAL

A MONTHLY REVIEW OF MEDICINE AND SURGERY.

*EDITORS:*

THOMAS LOTHROP, M. D. - - WM. WARREN POTTER, M. D.

All communications, whether of a literary or business nature, should be addressed to the managing editor: 284 Franklin Street, Buffalo, N. Y.

Vol. XXXIV. DECEMBER, 1894. No. 5.

### THE MISSISSIPPI VALLEY MEDICAL ASSOCIATION.

The twentieth annual meeting of the Mississippi Valley Medical Association, held at Hot Springs, Ark., November 20–23, 1894, marks an era in the medical society gatherings for the year. As the closing meeting for 1894, it furnishes the opportunity for us to make some general comments on the subject of such organizations, as well as some specific remarks in relation to this particular association.

Let us begin by calling attention to the fact that the organization of this body varies somewhat from the common. In the first place, an essential feature consists in the fact that nothing can be discussed during the sessions excepting science as it relates to the practice of medicine. All questions pertaining to the business conduct of the association are disposed of in committee apart from the regular sessions of the society ; furthermore, in its general conduct, business rules prevail and the foundation of all its work is that it considers scientific questions by day and devotes the hours of evening to social entertainments.

It is evident from the very nature of such a plan of organization, that strong men from all sections of the country are attracted to its meetings ; and in them, too, great advantages are obtained, not only to the members themselves, but the places where it meets feel its beneficial effects long after the gavel falls at the closing of the session.

For a great medical body like this to have had the audacity to appoint its annual meeting at Hot Springs, challenges the admiration and respect of the profession of the United States. Especially is this the case when we reflect that its meeting there was more than a success : it was a triumph. Several reasons conspired to make this meeting a marked one in the history of the association. Primarily, the president, Dr. Xenophon C. Scott, of Cleveland, proved an executive officer worthy of his trust. His well-known talent for the conducting of association meetings had been often brought into requisition before, but never was it tested more severely, nor did it ever prove more able to stand the strain than on this occasion. His annual address was a paper that merits the careful attention of physicians throughout the land.

Again, in the selection of Dr. Thomas E. Holland, of Hot Springs, as chairman of the committee of arrangements, the trust was committed to an able executive officer. To have organized successfully the medical profession of that resort in a way that brought into harmonious relation all legitimate resident practitioners and united the cordial support of the citizens of that American Carlsbad, gives evidence that no mistake was made in such selection. It was no easy task to bring into harmonious relationship the divergent interests and opinions of the people of that locality in regard to the feasibility of holding the meeting there, and to Dr. Holland's indefatigable energy is due the fact that so many members of the profession from all parts of the country were found in attendance.

A third feature, and one perhaps of the greatest moment in this relation, was the exceptional railway facilities that the association enjoyed. This is the first time in the history of medical meetings in this country when railways have given an all-around one-half fare to the members attending. In the present financial stringency it would have been impossible, perhaps, to have brought together a great number of medical men at such a remote point, had the ordinary rules of a fare of one and one-third rate prevailed. Furthermore, in running an official train of Pullman sleepers from St. Louis to Hot Springs and return, giving ample and comfortable accommodations to all who chose to avail themselves of its privileges, was a happy combination of comfort and pleasure. For these very exceptional railway privileges the association is indebted to the courtesy of Col. Harry C. Townsend, general passenger agent of the Missouri Pacific railway, and of his able coadjutor and lieutenant, Col. Charles E. Ware, both of whom gave their personal attention to the organization of the train and safely conducted it from start to finish. Both of these gentlemen remained with the association until its end and proved themselves not only splendid railway officials, but staunch personal friends of almost every man in attendance.

The social features of this meeting deserve brief comment. The official train reached Little Rock at 8 o'clock Monday morning, November 19th, and all were entertained at breakfast by Col. Townsend, after which carriages were provided by the physicians of Little Rock, in which a visit was made to all the points of interest. A reception and luncheon at the Capital hotel concluded the ceremonies at Little Rock.

Boarding the official train 2.45, Hot Springs was reached at 5 P. M., where carriages again met and conveyed its 220 travelers to the several hotels in that city. The visiting ladies were entertained by a local committee, headed by Mrs. T. E. Holland, who provided street car rides, carriage drives, donkey excursions and other delightful ways of visiting the points of interest. Besides this, receptions at the houses of prominent citizens, including Col. A. A. and Mrs. Woodhull, of the United States Army, a grand ball at the Arlington on Wednesday evening and a magnificent banquet at the Park Hotel on Thursday. These, together with numerous other and lesser ways of enjoyment, were provided with a lavish hospitality that seemed to know no end. The hotels, too, did everything possible to make their guests at home. Mrs. Lyman T. Hay, wife of the manager of the Arlington, was a hostess of which Hot Springs may well be proud.

The scientific character of the meeting demands brief notice. The papers read will soon be published in the journals and an abstract of the proceedings will also be printed with promptitude. Hence, our readers will be able to form a judgment upon this for themselves, but we venture to assert that in the main it will compare favorably with that of any other general medical meeting that has been held during the present year.

This association cannot be considered in any sense as a rival to the American Medical Association, but it is, nevertheless, a formidable contestant for scientific honors with that body, and, in our opinion, it is fast attaining a degree of excellence not only in quantity, but quality that will challenge the older and larger organization.

The officers for the ensuing year are: President, Dr. W. N. Wishard, of Indianapolis; vice-presidents, Dr. Thomas E. Holland, of Hot Springs, and Dr. Charles B. Parker, of Cleveland; secretary, Dr. F. C. Woodburn, of Indianapolis ; treasurer, Dr. George J. Cook, Indianapolis.

The next place of meeting is Detroit, Mich., where a royal welcome is sure to be extended.

## Figures and Tables

**Figure f1:**